# Slow Oscillation Amplitudes and Up-State Lengths Relate to Memory Improvement 

**DOI:** 10.1371/journal.pone.0082049

**Published:** 2013-12-04

**Authors:** Dominik P. J. Heib, Kerstin Hoedlmoser, Peter Anderer, Josef Zeitlhofer, Georg Gruber, Wolfgang Klimesch, Manuel Schabus

**Affiliations:** 1 Laboratory for Sleep, Cognition and Consciousness Research, Department of Psychology, University of Salzburg, Salzburg, Austria; 2 Division of Physiological Psychology, Department of Psychology, University of Salzburg, Salzburg, Austria; 3 Department of Psychiatry and Psychotherapy, Medical University of Vienna, Vienna, Austria; 4 Department of Neurology, Medical University of Vienna, Vienna, Austria; 5 The Siesta Group, Vienna, Austria; University of California, Riverside, United States of America

## Abstract

There is growing evidence of the active involvement of sleep in memory consolidation. Besides hippocampal sharp wave-ripple complexes and sleep spindles, slow oscillations appear to play a key role in the process of sleep-associated memory consolidation. Furthermore, slow oscillation amplitude and spectral power increase during the night after learning declarative and procedural memory tasks. However, it is unresolved whether learning-induced changes specifically alter characteristics of individual slow oscillations, such as the slow oscillation up-state length and amplitude, which are believed to be important for neuronal replay. 24 subjects (12 men) aged between 20 and 30 years participated in a randomized, within-subject, multicenter study. Subjects slept on three occasions for a whole night in the sleep laboratory with full polysomnography. Whereas the first night only served for adaptation purposes, the two remaining nights were preceded by a declarative word-pair task or by a non-learning control task. Slow oscillations were detected in non-rapid eye movement sleep over electrode Fz. Results indicate positive correlations between the length of the up-state as well as the amplitude of both slow oscillation phases and changes in memory performance from pre to post sleep. We speculate that the prolonged slow oscillation up-state length might extend the timeframe for the transfer of initial hippocampal to long-term cortical memory representations, whereas the increase in slow oscillation amplitudes possibly reflects changes in the net synaptic strength of cortical networks.

## Introduction

For a long time, sleep has been seen as a mere state of passiveness, which only serves resting and recovery purposes. Over centuries, sleep was a much studied topic, which even today appears to be a challenging research field with many unresolved mysteries. Many functions of sleep have been discussed, with sleep-dependent consolidation being a prevalent topic. According to the “active system consolidation” hypothesis, sleep actively strengthens and restructures memories and thereby promotes the consolidation of newly acquired memory traces [1].

It is believed that new, declarative knowledge is initially encoded in a fast-learning temporary memory storage as well as in a parallel slow-learning long-term memory storage, namely the hippocampus and the neocortex, respectively [2]. Studies using single-unit recordings in rodents have shown that after encoding, during quiet wakefulness or subsequent sleep, newly acquired memory traces become spontaneously and repeatedly reactivated. These reactivations are most prominent within the fast-learning hippocampus and are strongest during the occurrence of hippocampal sharp wave-ripple complexes [3,4,5]. During sleep, particularly during slow-wave sleep (SWS), hippocampal ripples - recorded in rats and humans - were found to be temporally linked to thalamo-cortically generated sleep spindles [6,7], which are proposed to support synaptic plasticity at the neocortical level [8]. 

According to the idea of an active system consolidation during sleep, hippocampal reactivations and sleep spindles are proposed to foster the gradual transformation of initially fragile hippocampal to permanent cortical long-term memory representations [1].

A key role in this model is assigned to the sleep slow oscillation. Slow oscillations are generated within cortical networks [9] and occur in human sleep electroencephalogram (EEG) with a mean spectral peak frequency of 0.7-0.8 Hz [10]. Every slow oscillation consists of a hyperpolarized “down”-state associated with a decreased and a depolarized “up”-state characterized by an increased cortical activity [11,12]. Besides cortical activity, also ripple and sleep spindle activity is strongly increased during depolarizing up-states of neocortical slow oscillations [13,14]. It is believed that the reactivation of newly learned hippocampal memories and moreover the transfer of these memories into the cortex is grouped by the up-state of the slow oscillation. Hence, according to this idea, reactivated hippocampal memories reach the cortex at a point in time in which most cortical neurons are depolarized and spindles are occurring. In other words, slow oscillations are proposed to synchronize and orchestrate a hippocampo-to-neocortical information transfer and hereby play a critical role in integrating newly acquired hippocampal memory traces into slow learning cortical long-term memory systems [1].

Indeed, inducing slow oscillations during SWS by transcranial direct-current stimulation (tDCS) [15] provided the first evidence for a causal role of slow oscillations in declarative memory consolidation. Besides increased overnight memory improvement, such stimulation also led to enhanced spindle activity and enhanced EEG power in the slow oscillation band. In line with these findings, several recent studies reported a positive correlation between the EEG power in the slow-wave frequency range (0.1-4 Hz) – often named slow-wave activity (SWA) – and overnight memory change in healthy children [16] and adults [17,18]. 

However, SWA does not contain information regarding the amplitude and/or the phase length of both the up-state and down-state of the slow oscillation. Using single slow oscillation events instead of SWA, Mölle and colleagues [19] showed that learning of a declarative word-pair task, compared to a non-learning control task, led to a specific amplitude increase in the up-state of the slow oscillation, whereas the down-state amplitude and the length of the slow oscillation were not affected. In this study, we address whether changes in certain slow oscillation features, such as slow oscillation peak amplitudes and phase lengths, are triggered in response to a declarative learning demand or are related to overnight gains or decrements in memory performance. Therefore, we re-analyzed a data set previously published by Schabus and colleagues [20].

## Materials and Methods

### Ethics Statement

The study was approved by the local research ethics committee (University of Salzburg, Ethics Committee) and was conducted in accordance with the ethical principles of the Declaration of Helsinki. Before participation, all subjects have given written informed consent.

### Subjects

For the current study, we (re-) analyzed a data set previously published by Schabus and colleagues [20]. This sample consisted of 24 healthy subjects (12 men) aged between 20 and 30 years (mean age=24.42 years, SD=2.59). All included subjects were healthy sleepers as assessed by a polysomnographic (PSG) screening night, questionnaires and wrist actigraphy. For a more detailed description, please refer to [20].

### Procedure

**Figure 1:**
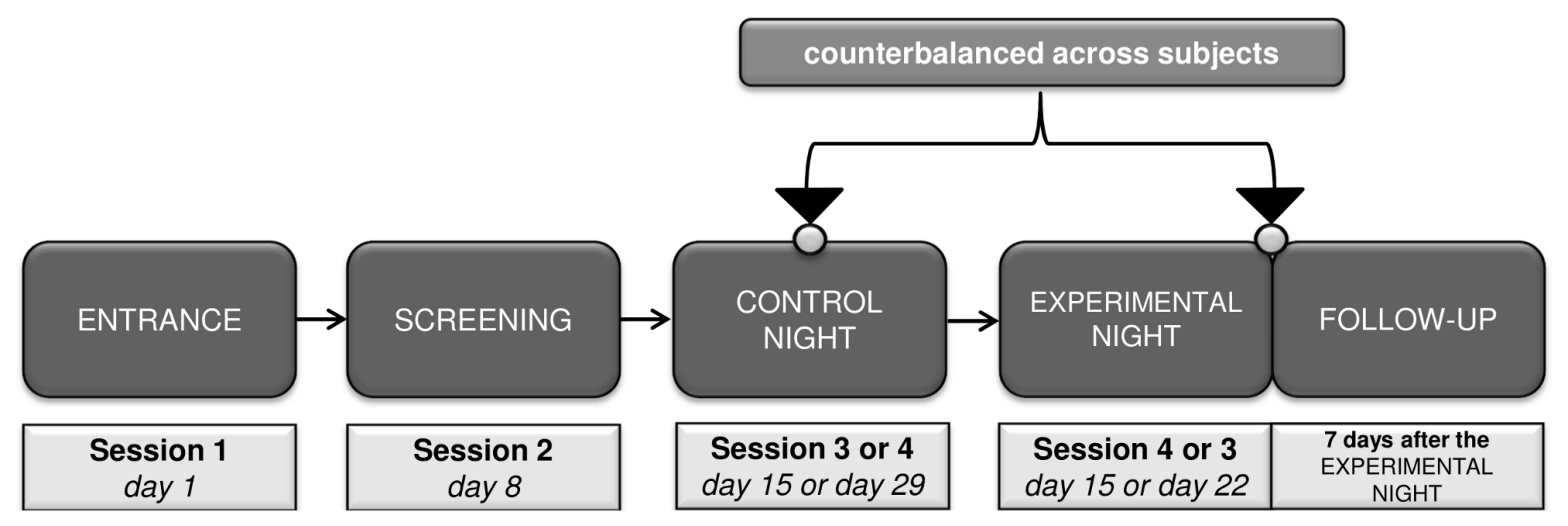
Study protocol. Depicted are the weekly laboratory sessions. Note that the CONTROL NIGHT was randomly scheduled either before or after the EXPERIMENTAL SESSIONS (EXPERIMENTAL NIGHT + FOLLOW-UP). FOLLOW-UP recall was only performed after the EXPERIMENTAL NIGHT.

Each subject completed five sessions in the sleep laboratory separated by 7 (± 1) days (cf. Figure 1). The first session served as an entrance examination in which subjects’ anamnesis as well as various psychometric tests (amongst others, the Advanced Progressive Matrices [APM [21]] and Wechsler Memory Scale-revised [WMS-R [22]]) were assessed [ENTRANCE].

In session 2 - 4, subjects slept for a whole night in the sleep laboratory with complete PSG. Whereas session 2 only served for diagnostic and adaptation purposes [SCREENING], session 3 and session 4 were either preceded (approximately 2.5 hours before sleep onset) by a declarative word-pair task [EXPERIMENTAL NIGHT] or by a non-learning control procedure [CONTROL NIGHT]. The order of CONTROL and EXPERIMENTAL NIGHT was counterbalanced across subjects. One week after the EXPERIMENTAL NIGHT, a follow-up testing of declarative memory performance was conducted [FOLLOW-UP]. 

PSG recordings started between 11:00 PM and midnight and were terminated after a subject’s habitual total sleep time or after 8 hours of time in bed.

### Task

#### Word-pair task

To investigate the effects of declarative memory learning on subsequent nocturnal sleep slow oscillation characteristics, subjects studied 160 word-pair associates prior to sleep.

The encoding session contained two learning blocks separated by a short break of approximately two minutes. In both blocks, each of the 160 word-pairs was presented once on a computer screen in white font on a black background. The order of word-pairs was randomized across subjects and learning blocks. 

After the encoding session, a first cued recall was conducted. The first word of each pair was presented as a retrieval cue and subjects were instructed to report the respective corresponding word. Recall performance was tested again in the morning after sleep (one hour after subjects were woken up) as well as in the morning one week after the learning session. For further details on task procedures please refer to [20]. The correct response score consisted of the number of correct responses and the number of (unambiguous) semantic correct answers (e.g., “flow” or “stream” instead of “river”), which were weighted by the factor 0.5. Recall performance is expressed as the percentage of correct responses (e.g. TEST1=[correct response score_TEST1_/160]*100). Accordingly, the overnight change in memory performance was defined as the difference between morning and evening recall performance.

#### Control task

The control task was highly similar to the experimental task, but lacking an intentional learning component. Visual input and task duration were equal to the word-pair task. 

The control task consisted of pseudo word-pairs in which size and font (italic/non-italic) of some letters were changed. In the control “encoding” session, subjects were instructed to silently count such deviating letters in each pseudo word-pair. In the control “retrieval”, subjects had to indicate the sum of deviant letters within each pseudo word-pair by pressing a respective response button. “Recall” performance (perceptual accuracy) is expressed as the percentage of correct button presses. Equally to the word-pair task, “recall” performance was tested before and after sleep and the change (here, perceptual) from evening to morning was calculated. Note that due to technical problems with the response pad, we are only able to report 17 perceptual performance values (seven missing, five males).

### Polysomnographic recordings and analysis

EEG was recorded using Synamps EEG amplifiers (NeuroScan Inc., El Paso, Texas). Twenty-two gold-plated silver electrodes (Fp1, FPz, Fp2, FCz, F7, F3, Fz, F4, F8, T3, C3, Cz, C4, T4, T5, P3, Pz, P4, T6, O1, Oz, O2 as well as A1 and A2 for later re-referencing) were placed on subjects’ scalps according to the 10-20 system [23]. Additionally, five electrooculogram (EOG) channels, one submental electromyogram (EMG) channel, one electrocardiogram channel (ECG) and one respiratory channel (chest wall movements) were recorded. During SCREENING, PSG setup consisted of eight EEG, four EOG, one ECG, three EMG (submental and left/right tibialis) and four respiratory channels (nasal airflow, chest and abdominal wall movements, oxygen saturation).

All signals were online referenced to FCz, digitized with 250 Hz sampling rate and filtered in a broad frequency band (0.10 Hz high-pass filter; 70 Hz low-pass filter; 50 Hz notch filter). Sleep was scored automatically according to standard criteria [24] (SOMNOLYZER 24*7; The Siesta Group ©) and verified manually by a sleep scoring expert.

**Figure 2:**
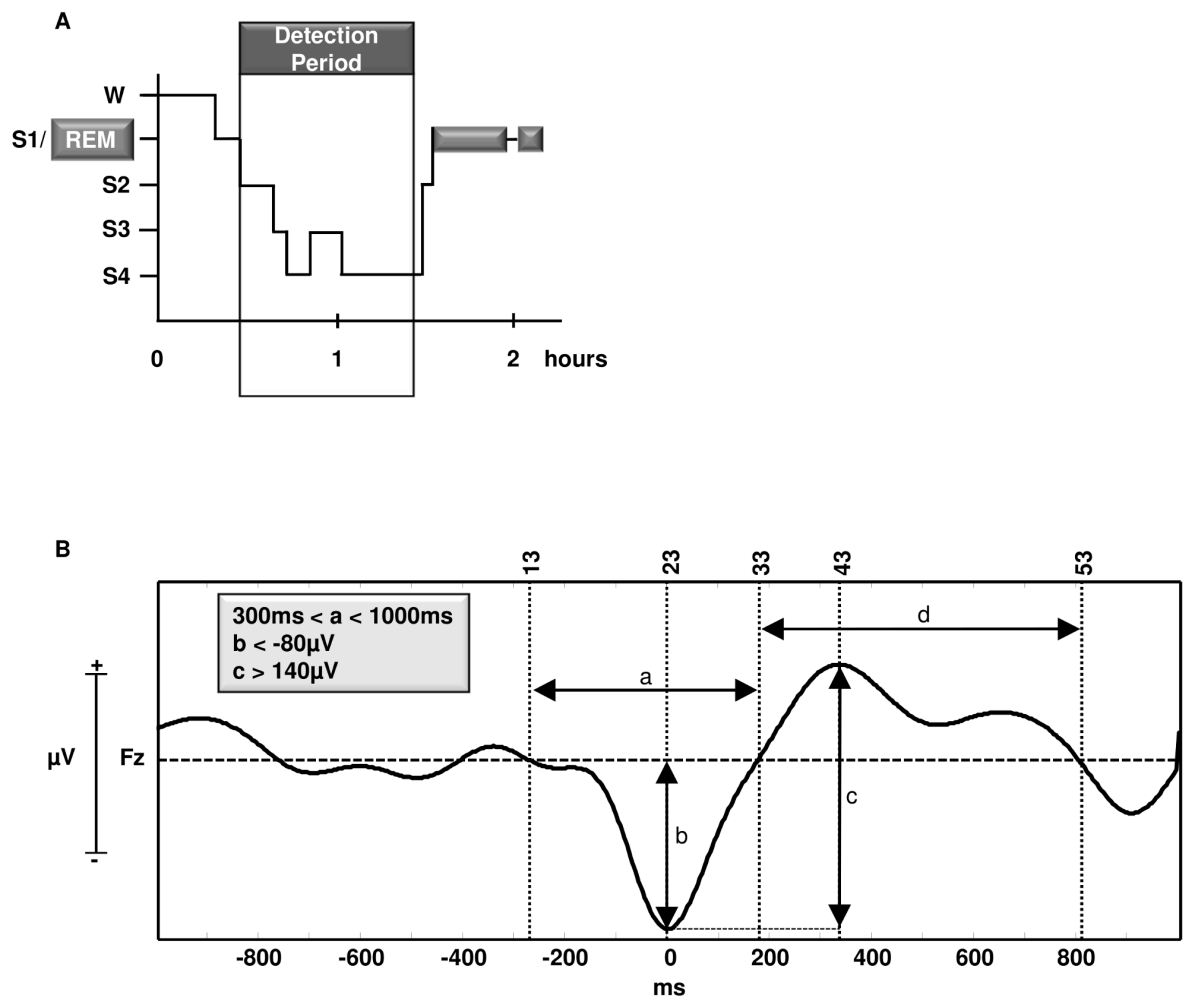
Automatic detection of slow oscillations. *A*, slow oscillations were detected from the time point at which subjects spent at least five minutes of continuous sleep in stage S2 or SWS and ended up to 60 minutes thereafter. *B*, generic detection of a slow oscillation in sleep stage S3. After low-pass filtering the signal at 4Hz, fixed detection criteria were applied to own-built Matlab routines (MathWorks®, Natick, MA). Each detection was specified by five time points: (13) start of the slow oscillation down-state (23), down-state peak (33), start of the up-state and end of the down-state (43), peak of the up-state (53), end of the up-state. a, down-state length; b, down-state peak amplitude; c, negative-to-positive peak-to-peak amplitude; d, up-state length.

After recordings were visually inspected for major artifacts, slow oscillations were detected over electrode Fz in the first hour of non-rapid eye movement sleep (NREM, S2-S4) starting with the first sleep epoch after which subjects spent at least five minutes of continuous sleep in stage S2 or SWS (cf. Figure 2a). At first, all signals were low pass filtered at 30 Hz, re-referenced to the average of A1 and A2, low pass filtered at four Hz and down-sampled to 100 Hz. Subsequently, to detect slow oscillations, standard detection criteria [25] were applied using own-built Matlab routines (MathWorks®, Natick, MA): (1) a positive zero crossing follows a negative zero crossing in a time window of 0.3-1s, (2) a peak negativity between such two zero crossings deceeds -80µV and (3) an amplitude difference between the peak negativity and a subsequent positive peak of at least 140µV. We restricted our analysis to the up-state (cf. Figure 2b, c-b) and down-state peak amplitude (cf. Figure 2b, b) as well as to the up-state (cf. Figure 2b, d) and down-state (cf. Figure 2b, a) length of detected slow oscillations. In one subject, slow oscillation detection (in both nights) had to be performed on electrode position F3, as signal quality on Fz (EXPERIMENTAL NIGHT) did not allow for any analyses. 

### Statistics

Statistical analyses were performed using PASW Statistics 18.0.2 software (SPSS Inc., Chicago, Illinois). Kolmogorov-Smirnov tests were applied to test for the normality of data distribution, which was given in all cases. The significance level was set to p<0.05. P-values<0.10 were reported as trends. 

Based on their overnight change in memory performance, subjects were divided into memory enhancers (I+; n=15) and memory non-enhancers (I-; n=9) using zero change as the cutoff value. Note that two subjects' recall performance was exactly alike during both evening and morning recall (zero change) and hence they were classified as memory non-enhancers.

Behavioral analyses were done using paired and independent-sample t-tests (2-tailed). Analyses on differences of slow oscillation characteristics between I+ and I- were performed using repeated-measures ANOVAs and post-hoc paired and independent-sample t-tests (2-tailed). For correlational analyses, 2-tailed Pearson’s bivariate as well as partial correlations were used.

## Results

### Behavioral analysis

Regardless of group affiliation (I+/I-), subjects correctly recalled 62.64% (SD=20.58%) of all word-pairs during the evening and 63.70% (SD=20.35%) on the subsequent morning. Overnight memory improvement closely failed to reach significance (t_23_=2.02, P=0.055). I+ showed a significant overnight gain in memory performance (t_14_=5.58, P<0.001), whereas I- significantly decreased their memory performance from evening to morning recall (t_8_=-4.35, P=0.002). Besides the difference in overnight memory change, I+ and I- did not differ in other (possibly confounding) variables, like IQ (APM), general memory capacity (WMS-R), absolute memory performance or age (all P>0.50, cf. Table 1).

**Table 1:** APM, WMS-R, age, and recall (evening, morning) performance for I+ and I-.

	**I-**		**I+**		
	**Mean**	**SD**	**Mean**	**SD**	**t22(P)**
APM	118.00	16.35	115.20	11.65	0.48 (0.64)
WMS-R	115.25	17.63	116.07	10.93	0.12 (0.89)
Age	24.00	2.51	24.67	2.77	-0.57 (0.58)
Evening recall	64.05	14.60	60.44	23.43	0.39 (0.70)
Morning recall	62.53	14.45	63.03	23.42	-0.06 (0.96)

The table depicts values for subjects’ mean IQ (APM), general memory capacity (WMS-R), age and recall (evening, morning) performance for I+ and I-. As revealed by independent-sample t-tests (2-tailed), I- and I+ did not differ in any of these parameters.

Concerning the control task, subjects’ average accuracy was 91.14% (SD=10.0%) during evening performance and 92.13% (SD=7.5%) on the next morning. Overnight improvement in perceptual accuracy failed to reach significance (t_16_=0.99, P>0.30). 

### Analysis on slow oscillations

To test whether declarative learning modifies slow oscillation characteristics (peak amplitudes, phase lengths) and whether such changes differ between I+ and I- , four 2-way ANOVAs with the within subject factor CONDITION (CONTROL vs. EXPERIMENTAL NIGHT) and the between subject factor MEMORY IMPROVEMENT (I+ vs. I-) were calculated. The dependent measure was either the up-state or down-state amplitude or the up-state or down-state phase length of the slow oscillation.

**Figure 3:**
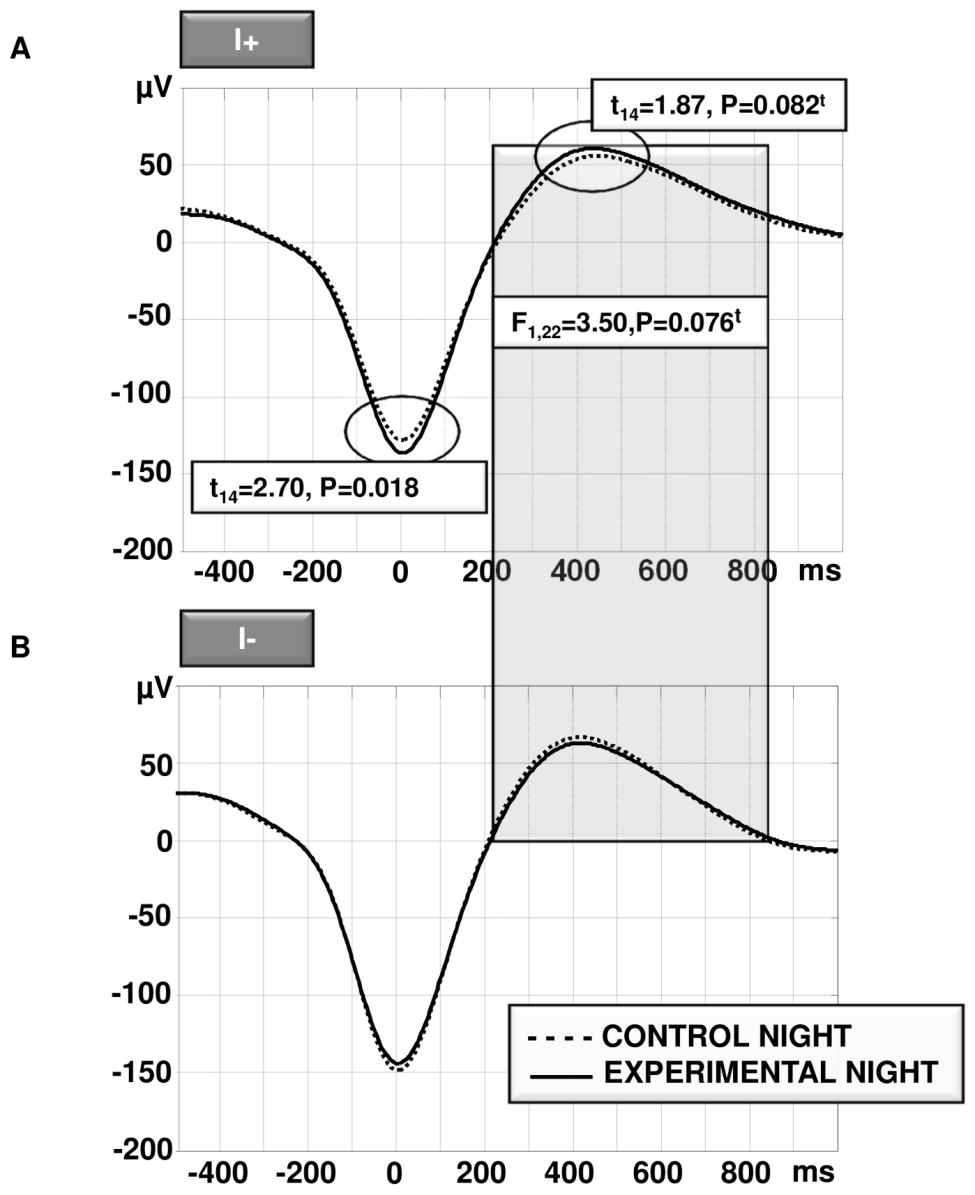
Learning induced changes in slow oscillations. *A*, subjects who increased their memory performance overnight (I+) also increased their down-state (P=0.018) and up-state peak (P=0.082^t^) amplitude from the CONTROL to the EXPERIMENTAL NIGHT. *B*, slow oscillations peak amplitudes did not differ between the CONTROL and the EXPERIMENTAL NIGHT in subjects who did not increase their memory performance from pre to post sleep (I-). As shaded, I+ and I- seem to generally differ in their up-state phase length (P=0.076^t^). t, statistical trend (P<0.10).

Regarding both up-state and down-state peak amplitude, there was no significant main effect for CONDITION nor for the between subject factor MEMORY IMPROVEMENT (all P>0.20). Thus, there was neither an overall change in peak amplitudes from the CONTROL to the EXPERIMENTAL NIGHT nor an overall difference between MEMORY IMPROVEMENT groups. However, we found a significant interaction between CONDITION*MEMORY IMPROVEMENT for both up-state (F_1,22_=5.78; P=0.025) and down-state peak amplitude (F_1,22_=5.44; P=0.029). Post-hoc tests revealed an enhanced down-state amplitude and a trend towards an increased up-state amplitude in the EXPERIMENTAL compared to the CONTROL NIGHT for I+ (down-state: t_14_=2.70, P=0.018; up-state: t_14_=1.87, P=0.082; cf. Figure 3a) and no change for I- subjects (all P>0.10; cf. Figure 3b). 

Concerning the length of slow oscillations, ANOVAs for both the up-state and the down-state length did not reveal a significant main effect for factor CONDITION nor for the interaction CONDITION*MEMORY IMPROVEMENT (all P>0.40). In other words, there was no general difference in the up-state or down-state length between the CONTROL and EXPERIMENTAL NIGHT and no differential change in phase lengths from the CONTROL to the EXPERIMENTAL NIGHT between I+ and I- subjects. However, results of the up-state length revealed a trend for the between subject factor MEMORY IMPROVEMENT (F_1,22_=3.50, P=0.076) indicating a generally more pronounced up-state length in I+ (cf. Figure 3, shaded area). 

To verify whether subjects’ IQ likewise interact with the change in the up-state length, we recalculated the respective ANOVA with IQ (APM) as the between group factor (subjects were divided into IQ+ [n=13] and IQ- [n=11] using the median as the cutoff value). Results revealed a significant CONDITION*IQ interaction (F_1,22_=5.91, P=0.024). However, as shown by post-hoc tests, neither IQ+ nor IQ- showed a significant change in the up-state length from the CONTROL to the EXPERIMENTAL NIGHT (all P>0.10). As opposed to the up-state length, neither the down-state length nor phase amplitudes revealed an interaction with subjects’ IQ (all p>0.50). It is important to note, that the described MEMORY IMPROVEMENT (I+, I-) effects are independent of subjects’ IQ as revealed by ANOVAs with IQ as the covariate (CONDITION*MEMORY IMPROVEMENT: down-state peak: F_1,21_=5.22, P=0.033; up-state peak: F_1_,_21_=5.44, P=0.033) and partial correlations (see below). 

**Figure 4:**
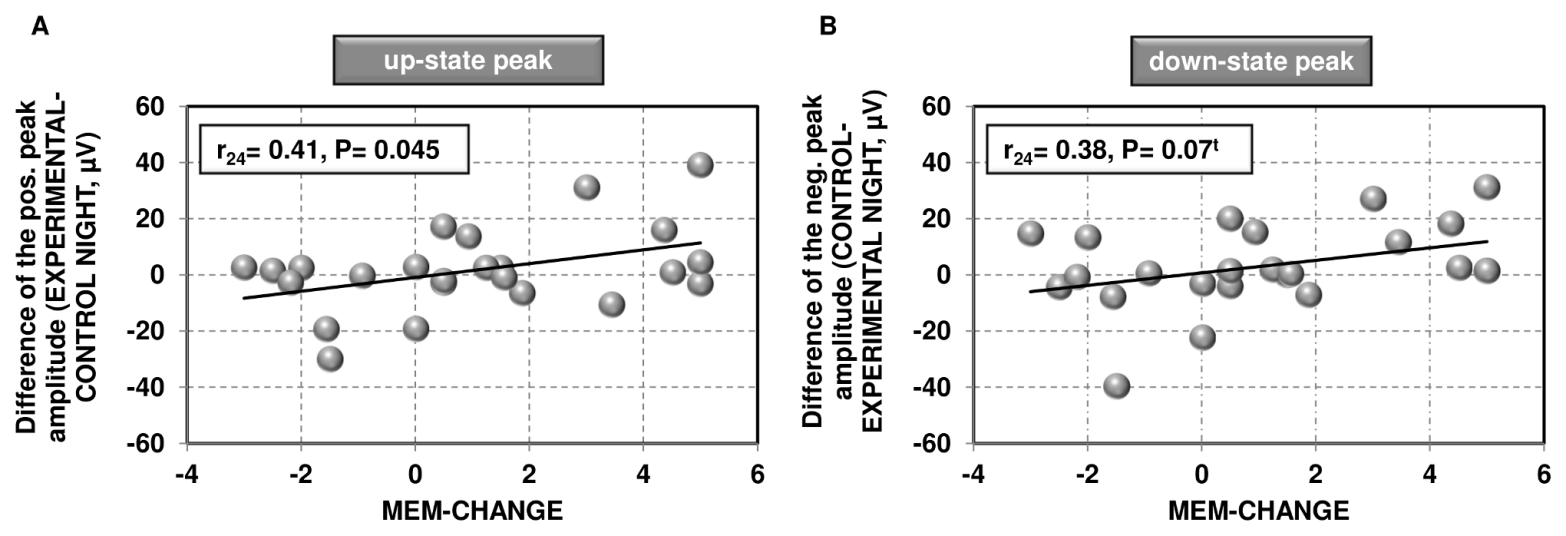
Relation between slow oscillation amplitudes and overnight change in memory performance. *A*, shown is a significant (2-tailed) correlation (P= 0.045) between changes in the slow oscillation up-state peak amplitude from the CONTROL to the EXPERIMENTAL NIGHT and the overnight change in recall performance. Note that subjects showing strong increases in their up-state amplitude are the ones showing the strongest overnight memory improvements. *B*, shown is a relation (P= 0.07^t^) between changes in the absolute down-state peak amplitude of the slow oscillation from the CONTROL to the EXPERIMENTAL NIGHT and the overnight change in recall performance. Note that subjects showing strong increases in their absolute down-state amplitude are the ones showing the strongest overnight memory improvements. t, statistical trend (P<0.10); MEM-CHANGE, overnight memory change (morning-evening recall performance).

Across group (I+/I-) analysis showed a significant positive relation between overnight changes in declarative memory performance and changes in the up-state peak amplitude of the slow oscillation from the CONTROL to the EXPERIMENTAL NIGHT (r_24_=0.41, P=0.045; cf. Figure 4a) and a trend towards a positive relationship between overnight memory change and changes in the absolute down-state peak amplitude (r_24_=0.38, P=0.070, cf. Figure 4b). When running partial correlations (2-tailed) controlling for subjects' IQ, these relationships were even stronger and both the change in the up-state (r_21_=0.44, P=0.037) and in the down-state peak amplitude (r_21_=0.42, P=0.048) were significantly related to overnight changes in memory performance.

**Figure 5:**
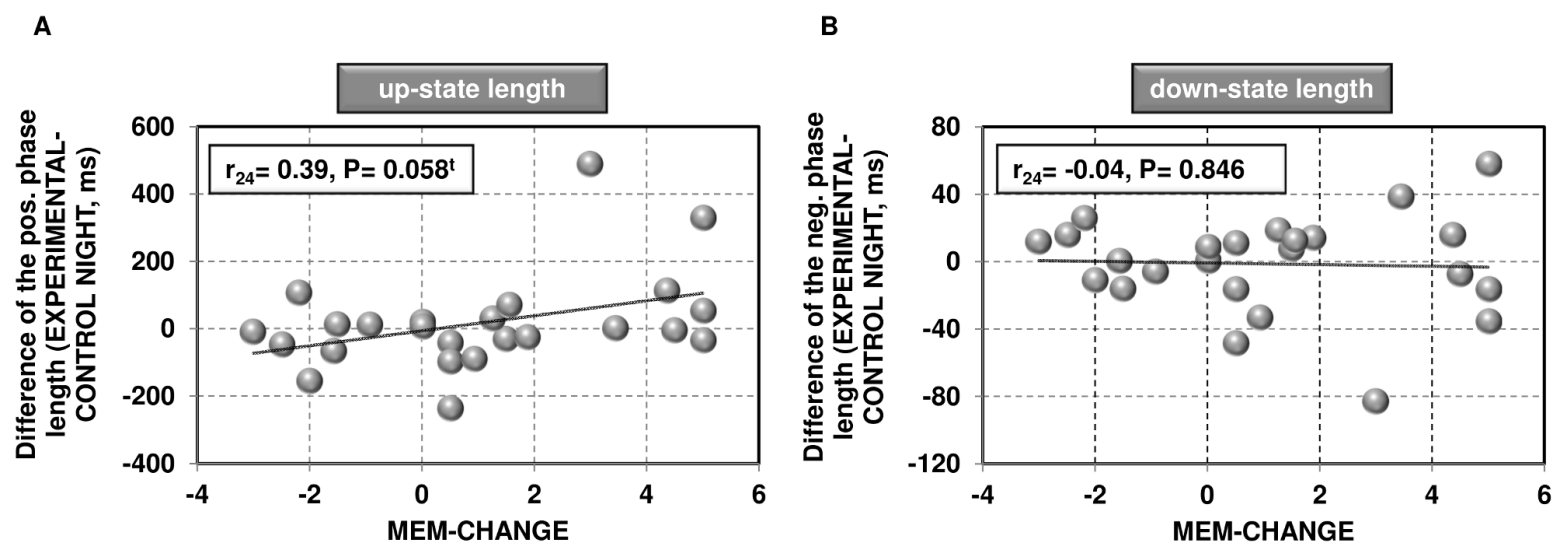
Relation between slow oscillation phase lengths and overnight change in memory performance. *A*, shown is the (2-tailed) correlation (P= 0.058^t^) between changes in the up-state length of the slow oscillation from the CONTROL to the EXPERIMENTAL NIGHT and the overnight change in recall performance. Note that subjects showing strong increases in their up-state phase length are the ones showing the strongest overnight memory improvements. *B*, shown is the zero-correlation between changes in the down-state length of the slow oscillation from the CONTROL to the EXPERIMENTAL NIGHT and the overnight change in recall performance. t, statistical trend (P<0.10); MEM-CHANGE, overnight memory change (morning-evening recall performance).

Furthermore, the correlation between the change in the up-state phase length from the CONTROL to the EXPERIMENTAL NIGHT and the overnight change in memory performance just failed to reach significance (r_24_= 0.39, P= .058; cf. Figure 5a). This relationship also persists when controlling for subjects’ IQ (r_21_=0.39, P=0.066). The length of the down-state was however not related to overnight changes in memory performance (r_24_=-0.04, P=0.846; cf. Figure 5b) 

The amount of overnight memory change was not related to traits, such as IQ (APM), general memory capacity (WMS-R), absolute memory performance or age (all P>.30). 

## Discussion

In this study it was investigated whether slow oscillation amplitudes and phase lengths are related to overnight changes in declarative memory performance. Our results demonstrate that whereas learning a declarative word-pair task prior to sleep does not alter (i) the up- or down-state peak amplitude or (ii) the up- or down-state length of the slow oscillation in general, there is a relation to individual overnight memory change.

Specifically, we revealed that the amount of overnight memory change is linearly related to induced changes in the up-state length as well as to changes in the up-state and down-state peak amplitude of the slow oscillation, which is in line with earlier reports relating slow oscillation power to changes in declarative [16,18] and procedural [17] memory performance from evening to morning. While all these results underline the role of slow oscillations for memory consolidation during sleep [26], we believe that, specifically, the separation of up- and down- states – and not merely the overall slow oscillation power – adds important information about state specific contributions to the consolidation process. Recent evidence clearly points to the fact that the depolarizing up-state of the slow oscillation is the more likely time window where information reprocessing occurs [26] or where incoming information during sleep is further processed [27,28]. We therefore argue that a relative increase in the length of the up-state phase might provide a longer time window for replaying and transferring new memories from the hippocampus into cortical networks. Consequently, the accessibility of newly stored memories should be facilitated and overnight memory change increased. 

Another prevalent hypothesis concerning learning-related slow oscillation changes has been postulated by Tononi and Cirelli [29,30]: the synaptic downscaling hypothesis. According to their view, learning during wakefulness leads to synaptic potentiation and slow oscillations - reflected by SWA - downscale this net increase in synaptic strength during SWS. As a result, exclusively strong memories survive this downscaling process and signal to noise ratio increases, which thus facilitate accessibility of these memory traces. The degree of synchronization between populations of neurons during sleep differs as a function of the synaptic strength at the end of the day. High synaptic strength leads to high rates of synchronization and as a consequence to high slow oscillation amplitudes. Hence, initial encoding efficiency or initial strength of newly acquired memory traces probably should separate memory enhancers (I+) from memory non-enhancers (I-) as only the former individuals show an increase of slow oscillation amplitudes following learning. However, in our study, I+ and I- did not differ in their absolute memory performance during recall before sleep. It can be argued that I+, irrespectively of the initial encoding strength, experience a stronger depotentiation during the night. This might be reflected by a relative increase in slow oscillation amplitudes and may lead to an increase in signal to noise ratio and consequently easier memory access the next day. 

Note, however, that there are also recent animal studies, which seem at odds with the assumptions of the synaptic downscaling hypothesis [31,32,33]. For instance, a study by Chauvette and collegues [31] revealed that long-term potentiation rather than downscaling of cortical networks occurs during SWS. Interestingly, in that study SWS-related potentiation was linked to hyperpolarization periods and thus establishes a link between slow-wave down-states, long-term potentiation and consequently also sleep-associated memory consolidation. In light of these findings, our results might therefore also reflect the efficiency of long-term potentiation processes during the night after learning a declarative memory task during the slow oscillation down-state. The exact significance of slow oscillation up- or down-states for long-term potentiation and memory consolidation is yet to be addressed.

Besides differences in learning induced changes, we also found a general difference regarding the up-state phase length of the slow oscillation between I+ and I-. Furthermore, changes in the up-state length were also related to subjects’ IQ. However, trait effects do not seem to significantly bias revealed memory change and slow oscillation effects. 

Learning induced changes in slow oscillation parameters were only evident in individuals who improved their memory overnight (cf. Figure 3). This implies that these modifications are not generally induced by learning. Regarding the length of the slow oscillation, our results resemble findings by Mölle et al. [19], as they also did not find general, learning induced modifications of the length of slow oscillations. Nevertheless, Mölle and colleagues could show a slight learning induced increase in the up-state amplitude at around 500-800ms after the peak of the down-state. As we only analyzed the peak amplitude of the up-state and as the up-state peak occurs most often before 500ms after the down-state peak, it is quite possible that point by point comparisons would have revealed a similar, general learning induced amplitude increase in such “late” timeframe of the slow oscillation up-state. Furthermore, we restricted our analysis to slow oscillations detected over site Fz. It is therefore possible that general learning induced changes in slow oscillation amplitudes and phase lengths could be revealed over other, probably more specifically task related, cortical regions. Last but not least, as this study was a first explorative study using different kind of slow oscillation measures, we did not apply any alpha-adjustment for multiple testing.

In conclusion, our data extend earlier findings and suggest that the length of the up-state of individual slow oscillations directly relates to overnight memory improvements in a declarative memory task. In addition, both the up-state and down-state peak amplitude were found to be related to overnight memory changes. We speculate that while a prolongation of the depolarizing up-state of the slow oscillation extends the time window for neuronal replay and therefore relates to overnight memory improvement, slow oscillation amplitude changes might reflect changes in the net synaptic strength of cortical networks. Such amplitude changes might either relate to synaptic downscaling and thus to an improved signal to noise ratio for better memory access or to a direct strengthening or integration of new memories within or into cortical networks by means of long term potentiation.
